# Biologics in Dermatology

**DOI:** 10.3390/ph6040557

**Published:** 2013-04-17

**Authors:** David Chandler, Anthony Bewley

**Affiliations:** 1 London School of Hygiene and Tropical Medicine, Keppel Street, WC1E 7HT, London, UK; 2 Whipps Cross University Hospital and the Royal London Hospital, Whitechapel, E1 1BB, London, UK; E-Mail: Anthony.Bewley@bartshealth.nhs.uk

**Keywords:** biologics, biological response modifiers, immunopathogenesis, dermatology, skin disease

## Abstract

Skin and subcutaneous diseases affect millions of people worldwide, causing significant morbidity. Biologics are becoming increasingly useful for the treatment of many skin diseases, particularly as alternatives for patients who have failed to tolerate or respond to conventional systemic therapies. Biological therapies provide a targeted approach to treatment through interaction with specific components of the underlying immune and inflammatory disease processes. This review article examines the increasing evidence base for biologics in dermatology, including well-established treatments and novel agents.

## 1. Introduction

“Biologics” or biological response modifiers have advanced the treatment of many inflammatory diseases, and their use in the treatment of psoriasis is well established. As our understanding of the immunopathogenesis of other skin diseases has increased, so has our interest in the use of biologic therapies. New applications for existing therapies are being recognised, and novel biologics agents are being developed. Biologics act by modifying the host response to disease and include monoclonal antibodies and a variety of protein drugs which alter the activity of cytokines (such as interferons and interleukins), enzymes and growth factors. Biological therapies may offer a more targeted approach to interrupting the underlying disease process, with fewer toxic side-effects, compared with the conventional systemic therapies. However treatment can be expensive, and their immunosuppressive properties may increase the risk of infections and cancer.

## 2. Psoriasis

Psoriasis is a chronic immune-mediated inflammatory skin disease. The prevalence of psoriasis in the UK is estimated at 1.3%–2.2% [1]. Worldwide, the epidemiology of psoriasis is poorly understood with reported population prevalence in adults ranging from 0.91% in the USA to 8.5% in Norway [2]. The reported prevalence of inflammatory arthritis among patients with psoriasis varies widely, with estimates from some studies as high as 30% [3]. The estimated prevalence of psoriatic arthritis in a UK population using the CASPAR criteria was 13.8% [4]. Conventional systemic therapies for moderate to severe psoriasis can be inadequate, and there is a risk of serious adverse effects and cumulative toxicity with long-term use. Patient dissatisfaction with these treatments is common. Biologic therapies offer the chance to improve disease control and reduce associated comorbidity. The British Association of Dermatologists Biologic Interventions Register (BADBIR) was created to evaluate long-term outcomes in these patients.

Several biologic agents have proved successful in the treatment of psoriasis. Currently four biologic agents are approved by National Institute for Health and Clinical Excellence (NICE) for use in moderate to severe chronic plaque psoriasis—infliximab, adalimumab, etanercept and ustekinumab.

The pathogenesis of psoriasis involves a complex interaction between environmental factors and genetic predisposition. Inflammatory dermal dendritic cells (iDDCs) produce IL-23, TNF and nitric oxide radicals, which promote the activation of T cells and contribute to plaque formation. Several studies have demonstrated the presence of discrete populations of Th1 and Th17 cells in psoriatic skin lesions, confirming the significance of T-cell mediated immune responses in the disease process [5]. Many of the genes identified as conferring susceptibility to psoriasis belong to the IL-23-Th17 axis, the NF-kB pathway and the epidermal differentiation complex [6].

### 2.1. TNF Antagonists

The tumour necrosis factor (TNF) antagonists include infliximab and adalimumab (anti-TNF monoclonal antibodies) and etanercept (a human TNF receptor 2 Fc fusion protein). All three agents bind to both soluble (sTNF) and transmembrane (tmTNF) forms of TNF, interrupting TNF-mediated inflammatory pathways and inducing tmTNF reverse signalling.

Short-term data suggests that etanercept and infliximab can significantly improve symptoms of psoriatic arthritis and delay disease progression. Minor adverse events are common although further research is needed to review the long-term benefit and safety profile of these agents [7,8]. Recent evidence from a randomised, double-blind, placebo-controlled trial has demonstrated superior efficacy of combination treatment with etanercept plus methotrexate over etanercept monotherapy, with no increase in the rate of serious adverse events [9]. TNF antagonists have been shown to produce significant improvement in symptoms, health-related quality of life and functional status. In a phase IIIB open-label trial (PROGRESS) [10] patients treated with adalimumab for 16 weeks reported improvement in sleep quality and other outcomes including work productivity, daily activity and disease-associated pain.

Results from a recent phase II RCT show that treatment with certolizumab pegol (CZP) produces significant clinical benefit at week 12 in patients with moderate to severe plaque psoriasis [11]. PASI 75 responses were achieved by 75%, 83% and 7% of patients in the CZP 200 mg, CZP 400 mg and placebo groups, respectively. Furthermore, 47% of patients in the CZP 400 mg group achieved a PASI 90 response at week 12, which is better than that of adalimumab and etanercept. Patients in the CZP treatment groups also demonstrated clinically meaningful improvements in health-related quality of life (HRQoL) as measured by DLQI. CZP is a PEGylated anti-TNF agent, with a molecular structure different to that of the other TNF antagonists. It is composed of a single Fab of human anti-TNF conjugated with polyethylene glycol (PEG). It lacks an Fc portion, and the mechanism of action therefore does not involve antibody-dependent cellular cytotoxicity, complement activation or apoptosis in T cells or macrophages [12].

Capillary dilatation and angiogenesis are recognised features of many inflammatory conditions, including psoriasis. Recent experimental data have demonstrated superior clinical and histological improvement with a novel bispecific anti-TNF-Ang2 antibody compared with anti-TNF antibody alone, in a TNF transgenic mouse model of arthritis. Angiopoietin 2 (Ang2) is a growth factor which binds the TIE-2 tyrosine kinase receptor to promote angiogenesis and cause endothelial destabilisation. These results highlight the potential of treatments that target specific components of the angiogenesis pathway for the treatment of chronic inflammatory conditions [13]. Results of subsequent research in this area are encouraging. “Valpha” is a chimeric “decoy receptor” which simultaneously binds vascular endothelial growth factor (VEGF-A) and TNF-α. Increased expression of VEGF-A is noted in the skin lesions and serum of patients with psoriasis, and VEGF-related gene polymorphisms are known to contribute to psoriasis susceptibility. In a mouse model of psoriasis, Valpha produced significant improvements in epidermal hyperplasia as well as normalising blood and lymphatic vessel abnormalities [14,15].

### 2.2. Interleukin Antagonists

Ustekinumab is a fully human IgG1k monoclonal antibody with high affinity and specificity for the common p40 subunit shared by interleukin (IL)-12 and IL-23. Increased levels of IL-23 and Th17 cytokines have been detected in human psoriatic skin and IL-23 has been shown to induce psoriasis-like skin inflammation in mice [16]. Ustekinumab has been successful in the treatment of moderate to severe plaque psoriasis, with superior efficacy over etanercept demonstrated in the ACCEPT trial [17]. Ustekinumab has also been shown to achieve sustained clinical response with a favourable safety profile for up to four years of treatment in the PHOENIX 1 and 2 trials [18].

Recognition of the importance of Th17 cell responses in the pathogenesis of psoriasis has led to increasing focus on therapies that target IL-17, with promising results. A recent phase II RCT compared brodalumab (AMG-827, a humanised anti-IL-17-receptor monoclonal antibody) with placebo in patients with severe plaque psoriasis. Clinically and statistically significant improvements were noted in the intervention group, with greater improvement seen at higher treatment doses. Two patients treated with a higher dose of brodalumab (210 mg) developed reversible neutropenia, and no other serious adverse effects were noted [19]. Ixekizumab (a human anti-IL-17 monoclonal antibody) was evaluated in patients with moderate to severe chronic plaque psoriasis in a recent placebo-controlled trial. Significant clinical improvement was seen from as early as week one with sustained responses throughout the 20 week treatment period. A dose-dependent response was noted, with over a third of patients in the highest dose group (150 mg) achieving a 100% reduction in PASI score [20]. Neutropenia was observed in two patients. Secukinumab is another anti-IL-17A monoclonal antibody that has demonstrated efficacy for induction and maintenance treatment in patients with moderate to severe psoriasis. Further studies are needed to assess the long-term efficacy and safety profile for these treatments.

### 2.3. Protein Kinase Inhibitors

An exciting development in the treatment of psoriasis involves targeting Janus kinase (JAK) signalling. Preclinical data have identified cytokine signalling through the JAK-STAT pathway as a major component in the pathogenesis of psoriasis. Tofacitinib, a novel oral JAK inhibitor, disrupts cytokine signalling through inhibition of JAK1 and JAK3 leading to altered lymphocyte function. A recent phase II RCT compared treatment with oral tofacitinib *versus* placebo over 12 weeks in patients with moderate to severe plaque psoriasis [21]. Significant clinical improvement was noted in the intervention group from week 2 with sustained efficacy to week 12. No serious adverse events were noted.

Protein kinase C (PKC) has a key role in regulation of immune cell function. Sotrastaurin, a maleimide-based PKC inhibitor, is thought to block early activation of T-lymphocytes and is currently under investigation for the prevention of transplant rejection and the treatment of autoimmune and inflammatory conditions including psoriasis. *In vitro* experiments have shown that sotrastaurin blocks the production of pro-inflammatory cytokines by activated T-cells, macrophages and keratinocytes. Subsequent clinical and histological improvement has been reported in patients with moderate to severe plaque psoriasis within two weeks of starting treatment [22].

### 2.4. Other

Apremilast (CC-10004) is a novel biologic agent that specifically targets phosphodiesterase 4 (PD-4). Phosphodiesterase 4 is an intracellular enzyme that is expressed predominantly in immune cells, including dendritic cells, neutrophils and monocytes, but also in keratinocytes. PD-4 causes degradation of the secondary messenger cyclic AMP (cAMP) leading to increased production of pro-inflammatory mediators such as TNF-α, interleukins 2, 12 and 23, and chemokine (C-X-C motif) ligands 9 (CXCL9) and 10 (CXCL10) [23]. Therefore inhibition of PD-4 by apremilast has the potential to reduce pro-inflammatory cytokine production and subsequent inflammatory signalling. A recent phase II RCT was carried out to assess the efficacy of apremilast for moderate to severe psoriasis [24]. Patients were randomised to receive placebo or apremilast at 10, 20 or 30 mg twice daily over 12 weeks. The primary endpoint was the proportion of patients with a PASI 75 response at week 16. This was achieved in 6% of placebo patients and 11%, 29% and 41% of patients assigned apremilast at 10, 20 and 30 mg, respectively. Apremilast appears to be efficacious and tolerable at doses of 20 or 30 mg twice daily, and to date there is no evidence of serious adverse events. Further investigation of the safety profile with long-term use is needed, and a phase III trial using apremilast at 30 mg twice daily is in progress.

Efalizumab is a recombinant humanised monoclonal antibody against the CD11a subunit of the cell surface protein LFA-1 (lymphocyte function-associated antigen-1). Binding of LFA-1 to intracellular adhesion molecule (ICAM) 1–3 is an important step in the pathogenesis of psoriasis, causing activation, and migration of T-lymphocytes into the skin. Studies have also shown efalizumab to downregulate several other T-cell surface molecules. Unfortunately three cases of confirmed progressive multifocal leukoencephalopathy (PML) have been reported in patients who had received the treatment for three years or more. This has resulted in the treatment being withdrawn from use.

## 3. Autoimmune Blistering Disorders

Pemphigus describes a group of uncommon autoimmune mucocutaneous blistering disorders, which can be fatal if left untreated. The most common type of pemphigus is pemphigus vulgaris (PV), although many other less common variants exist. The pathogenesis of PV involves circulating immunoglobulin G (IgG) autoantibodies against desmogleins 1 and 3 (desmosomal cadherins involved in epidermal intercellular adhesion). Conventional treatments include systemic corticosteroids, immunosuppressive and anti-inflammatory agents. Biologic agents are able to target specific pathways in the pathogenesis of the disorder, and have been used effectively in the treatment of PV.

Rituximab is a chimeric human/murine IgG1 monoclonal antibody against the CD20 protein expressed on the surface of B-lymphocytes. Rituximab targets pre-B and mature B lymphocytes causing complement and antibody-dependent cytotoxicity and apoptosis thus preventing their development into antibody-producing plasma cells. The CD20 protein is not expressed on the surface of terminally differentiated plasma cells. Studies have shown rituximab to be an effective and safe treatment for PV at a dose of 4 × 375 mg/m^2^, as currently approved for the treatment of B-cell malignancies, and more recently at the higher dose approved for the treatment of rheumatoid arthritis [25,26]. In a study of 23 patients with severe pemphigus, treatment with a combination of immunoadsorption, rituximab, pulsed dexamethasone and azathioprine/mycophenolate mofetil resulted in rapid improvement of pemphigus lesions and complete remission in 83% of patients throughout the duration of the study. This clinical improvement was paralleled by a sudden and prolonged reduction in the levels of circulating desmoglein-specific antibodies [27]. Case reports have described the effectiveness of the TNF-α antagonists infliximab and etanercept in the treatment of severe pemphigus [28,29] however clinical trials are lacking.

## 4. Hidradenitis Suppurativa

Hidradenitis suppurativa (HS) is a chronic inflammatory skin disease affecting the apocrine sweat glands, in intertriginous areas such as the axillae and groins. The pathophysiology is thought to involve follicular apocrine occlusion with subsequent perifolliculitis. Clinical presentation is highly variable and often recurrent, ranging from tender papules and pustules to painful deep-seated nodules with abscesses, sinus tract formation and scarring. Studies have highlighted components of immune dysregulation in HS that support the use of biologic agents that target TNF-α, IL-1β and IL-10 [30,31] The success of the TNF-α antagonists in the treatment of HS is documented in many case reports, and recent clinical trials provide more robust evidence for the efficacy and safety of treatment with adalimumab at 40 mg per week [32,33]. A recent systematic review of TNF-α antagonists for severe hidradenitis suppurativa supports the use of both infliximab and adalimumab, but also highlights the lack of available high-quality evidence [34]. In a retrospective study comparing adalimumab with infliximab for the treatment of severe HS, infliximab was shown to produce a greater reduction in disease severity as measured by the Sartorius scoring system [35]. The effectiveness of the TNF-α antagonists has stimulated interest in understanding the immune-pathogenesis of this condition, with recent evidence identifying the potential importance of the IL-23/Th17 pathway. Lesional skin samples from 10 patients with HS demonstrated increased expression of IL-12 and IL-23 by dermal macrophages and a prominent infiltration of Th17 cells when compared with healthy skin [36]. Biologic agents such as ustekinumab that target the p40 sub-unit common to IL-12 and IL-23 could therefore offer an effective treatment option for patients with HS [37]. There are reported cases of HS responding to treatment with ustekinumab, and further investigation is warranted.

## 5. Pyoderma Gangrenosum

Pyoderma gangrenosum (PG) is an uncommon neutrophilic dermatosis, in which sterile nodules or pustules break down to form progressively enlarging ulcers with undermined violaceous borders. It is associated with systemic disease, most commonly inflammatory bowel disease, in approximately half of cases. The aetiology is poorly understood however dysregulation of the immune system, specifically altered neutrophil chemotaxis, is thought to be important [38]. Management of this condition can be challenging, and systemic immunosuppressive therapies are often required. There is a lack of high quality evidence regarding the commonly used systemic treatments, and a randomised controlled trial (RCT) comparing ciclosporin to prednisolone (STOP GAP trial) is currently in progress [39]. The TNF-α antagonists have been used effectively in the treatment of PG. In one small RCT patients received a single infusion of infliximab at 5 mg/kg or placebo. Response rates (clinical improvement) at two weeks were 46% and 6% in the infliximab and placebo groups, respectively. Small case series have demonstrated rapid clinical improvement in patients with PG associated with Crohn’s disease [40,41]. Several case reports describe the successful treatment of PG with etanercept and ustekinumab. In one case report, tissue sample analysis from a PG lesion demonstrated an elevated expression of IL-23, and the authors proposed a role for IL-23 in the pathogenesis of PG through the stimulation of IL-17-mediated inflammation and neutrophil recruitment, similar to that of psoriasis. Subsequent treatment with ustekinumab resulted in sustained clinical improvement and reduced expression of IL-23 [42]. Randomised controlled trials are required to assess the role of the various biologic agents in the treatment of pyoderma gangrenosum, and inform clinical decision-making.

## 6. Skin Cancer: Malignant Melanoma

The incidence of cutaneous malignant melanoma has increased progressively over the last 50 years [43]. In 2010, over 12,000 new cases of malignant melanoma were diagnosed in the UK. Dacarbazine remains the standard therapy for metastatic melanoma, however objective tumour response and survival rates with this treatment are unsatisfactory. Various immunotherapies are now available for the treatment of malignant melanoma, and our increasing knowledge of the biological processes underlying melanoma has allowed the development of novel targeted biologic agents.

### 6.1. Interferon

Interferon alpha (IFN-α) has various mechanisms of action in many malignancies, including anti-angiogenic, immunoregulatory, differentiation-inducing, anti-proliferative and pro-apoptotic effects [44]. It is known to promote a Th1 shift in host immunity against tumours and contribute to the development of anti-tumour immunity through effects on dendritic cells. The use of high-dose IFN-α2b as adjuvant therapy for high-risk surgically resected melanoma (stage IIB or III) in the eastern cooperative oncology group (ECOG) and intergroup trials produced sustained improvement in relapse-free survival [45]. Improvement in relapse-free survival has also been demonstrated with the use of high-dose adjuvant pegylated IFN-α2b, in the European Organisation for Research and Treatment of Cancer (EORTC) trial, however this benefit was confined to a subset of patients with microscopic nodal disease [46].

### 6.2. Interleukin (IL)-2

The efficacy of high-dose intravenous recombinant interleukin (IL)-2 for patients with metastatic melanoma was assessed using data from 270 patients treated between 1985 and 1993 [47]. A sustained complete response was seen in 6% of patients, however significant morbidity occurred due to drug toxicity. Further studies are needed to identify clinical or biological markers of therapeutic predictive value and allow targeted treatment to those patients most likely to respond. Adoptive cell therapy with autologous tumour infiltrating lymphocytes (TIL), following chemotherapy induced lymphodepletion, together with high-dose IL-2 has produced high sustained response rates in patients with advanced malignant melanoma, [48] although significant IL-2 associated toxicity has been observed. A recent pilot trial of six patients has demonstrated that it is possible to induce complete response to treatment with adoptive cell therapy in combination with low-dose IL-2 [49]. This treatment was better tolerated, however larger clinical studies are needed to ascertain if the response to low-dose IL-2 can be maintained.

### 6.3. Anti-CTLA-4 Monoclonal Antibodies

Cytotoxic T-Lymphocyte Antigen 4 (CTLA-4) or CD152 is a protein receptor expressed on the surface of helper T cells. Binding of CD80 and CD86 (B7-1 and B7-2 respectively) on antigen presenting cells to the co-stimulatory T cell surface receptor CD28 results in the transmission of a stimulatory signal to T cells leading to T cell activation. Conversely, activation of CTLA-4 by CD80 and CD86 transmits an inhibitory signal to T cells, with downregulation of IL-2 gene transcription and reduced T-cell proliferation.

Ipilimumab is a fully human monoclonal antibody directed against CTLA-4. By binding to CTLA-4, ipilimumab interrupts the transmission of B7-CTLA-4-mediated inhibitory T cell signalling, resulting in unopposed activation of T cells and stimulation of anti-tumour immune responses [50]. The results of a multi-centre clinical trial [51] showed that combination treatment with dacarbazine plus ipilimumab produced improved overall survival compared with dacarbazine plus placebo (11.2 months *vs.* 9.1 months, respectively), in patients with previously untreated metastatic melanoma. Similar results were seen in a three-arm, multi-centre, randomised, double-blind trial, [52] in patients with unresectable stage III or IV melanoma whose disease had progressed whilst receiving treatment for metastatic disease. Ipilimumab, given with or without the gp100 peptide vaccine, improved overall survival compared with those who received the vaccine alone (median overall survival of 10.1 and 10.0 months *vs.* 6.4 months respectively, *p* ≤ 0.003).

Tremelimumab is another human (IgG2) monoclonal antibody against the CTLA-4 T cell receptor. Phase I and II clinical studies of tremelimumab have shown it to have an acceptable safety profile, and evidence of sustained anti-tumour activity [53,54,55]. However when tremelimumab was compared with standard single-agent chemotherapy (temozolomide or dacarbazine) as a first-line treatment in patients with unresectable stage III or IV melanoma, no difference in overall survival was detected [56].

### 6.4. BRAF Inhibitors

The identification of “activating mutations” in genes that control tumour proliferation has allowed the development of targeted biologic agents. Mutations have been detected in BRAF, NRAS and c-KIT which lead to activation of the RAS/RAF/MEK/ERK pathway and dysregulation of melanoma cell proliferation. The most frequently mutated of these genes is BRAF, with substitution of glutamic acid for valine at position V600 being the most common mutation [57].

Vemurafenib is an oral selective inhibitor of oncogenic V600E mutant BRAF. It is indicated as monotherapy for the treatment of V600E mutation-positive unresectable or metastatic melanoma. The interim analysis results from a phase III randomised multi-centre trial (BRIM-3) [58,59] showed significant improvement in overall survival and progression-free survival with vemurafenib compared to dacarbazine (relative risk reductions of 63% and 74% respectively, *p* < 0.001), in patients with previously untreated V600E BRAF mutation-positive metastatic melanoma. Vemurafenib is associated with a high risk of cutaneous adverse events (particularly rash, photosensitivity, pruritus and squamoproliferative eruptions) although the evidence from clinical trials suggests that these are manageable and permanent treatment discontinuation is unlikely to be necessary [57].

Dabrafenib is another oral selective BRAF inhibitor which has demonstrated clinical efficacy with an acceptable safety profile in phase I and II trials. In a recent phase III randomised controlled trial (BREAK-3), [60] dabrafenib significantly improved progression-free survival compared with dacarbazine (median 5.1 months and 2.7 months respectively, *p* < 0.0001), in patients with previously untreated stage IV or unresectable stage III V600E BRAF mutation-positive melanoma. Dabrafenib is characteristically associated with induction of keratinocyte proliferation, which can produce a range of clinical manifestations from benign seborrhoeic keratosis to malignant well-differentiated squamous cell carcinoma (SCC) [61].

BRAF inhibitors have also produced clinical benefit in patients with the less common V600K and V600R mutations [62]. In patients with sensitive V600E/K/R BRAF mutations, RAF inhibitors block the mitogen-activated protein kinase (MAPK) signalling pathway and decrease tumour growth. However, in resistant RAS/RAF wild-type tumours, RAF inhibitors paradoxically activate the MAPK pathway leading to enhanced proliferation and migration of melanoma cells [63,64].

### 6.5. MEK Inhibitors

Trametinib is an oral selective inhibitor of MEK 1 and 2 (or MAP2K 1 and 2), both of which are types of mitogen-activated protein kinase kinase (MAP2K). Inhibition of these dually specific threonine/tyrosine kinases leads to down-regulation of the RAS/RAF/MEK/ERK signalling pathway. In a phase III open-label clinical trial (METRIC) [65] trametinib was shown to significantly improve rates of progression-free and overall survival compared with chemotherapy with dacarbazine or paclitaxel (6 month overall survival of 81% and 67% respectively, patient crossover included, *p* = 0.01), in patients with V600E or V600K BRAF mutation-positive metastatic melanoma.

Treatment with combined BRAF and MEK inhibition in V600 BRAF mutation-positive metastatic melanoma has been evaluated in phase I and II clinical trials [66]. The combination of dabrafenib and trametinib at full monotherapy doses has an acceptable safety profile, with the most common adverse events being pyrexia, fatigue and dehydration. BRAF inhibitor-induced hyperproliferative skin lesions occurred with lower frequency than with monotherapy. Median progression-free survival in the combination group was 9.4 months, compared with 5.8 months in the group treated with dabrafenib monotherapy (*p* < 0.001). Phase III trials are currently in progress to further evaluate the potential of this combination treatment (ClinicalTrials.gov Identifiers: NCT01584648, NCT01597908).

Sorafenib is an oral “multi-kinase” inhibitor that exhibits a range of antineoplastic effects through the inhibition of several protein kinases including the Raf serine/threonine kinases (C-Raf and B-Raf) and the receptor tyrosine kinases which include the vascular endothelial growth factor receptors (VEGFR 1, 2 and 3), platelet-derived growth factor receptor (PDGFR), c-Kit (DC117) and FLT3 (CD135) [67,68]. The use of sorafenib as a monotherapy does not appear to be efficacious, and the results of a large phase III trial [69] of sorafenib in combination with the chemotherapeutic agents carboplatin and paclitaxel have failed to demonstrate any benefit in progression-free and overall survival compared to the chemotherapy alone [70]. Large phase II studies evaluating the use of sorafenib in combination with dacarbazine [71] and temozolomide [72] have demonstrated clinical activity against advanced melanoma with acceptable safety profiles, however further studies are required to confirm these findings.

Molecular studies have demonstrated that MEK inhibition stimulates increased invasiveness of melanoma cells. The mechanism of invasion is dependent on integrin-mediated adhesion, a process which is heavily regulated by SRC kinases [73]. The combination of the MEK inhibitor selumetinib and the SRC kinase inhibitor saracatinib has been shown to suppress growth and invasion of melanoma cells *in vitro*. However in a phase II clinical trial [74] oral saracatinib monotherapy in 23 patients with advance melanoma failed to produce any objective clinical response, although there was evidence of inhibited T cell function and reduced cytokine (IL-2) production.

### 6.6. Other

Another mechanism through which melanoma may evade the host immune response is through the interaction between programmed cell death protein 1 (PD-1) and the programmed cell death 1 ligands PD-L1 and PD-L2 (B7-H1 and B7-DC respectively). PD-1 is a cell surface membrane protein belonging to the immunoglobulin superfamily, and is expressed on the surface of T cells, B cells and macrophages [75]. PD-1 and its ligands negatively regulate the immune system, causing immune tolerance through apoptosis of activated lymphocytes [76,77]. MDX-1106 and MDX-1105 are fully human IgG4 monoclonal antibodies specifically targeting PD-1 and PD-L1, respectively. Early phase clinical trials have shown treatment with these novel therapies to be well tolerated and capable of inducing durable anti-tumour activity [78,79].

## 7. Granulomatous Disease: Cutaneous Sarcoidosis

Sarcoidosis is multisystem inflammatory disease, characterised by the formation of non-caseating granulomas. Skin involvement is variable and occurs in approximately 25% of cases [80]. Observed immune responses involve CD4+ T lymphocytes and dermal dendritic cells, with the production of predominantly Th1 and Th17 cytokines [81]. TNF-α in particular is known to participate in the induction and maintenance of granulomas, and high levels seem to correlate with disease progression [82]. TNF-α blockade is an established therapeutic strategy for sarcoidosis, however there are no trials comparing efficacy among the different TNF-α antagonists. Evidence from several case series [83,84,85] support the use of infliximab and adalimumab in the treatment of cutaneous sarcoidosis, particularly in those patients with corticosteroid-refractory disease, however controlled clinical trials are needed to characterise the efficacy, optimal doses and safety profiles of these agents in clinical practice. There are case reports [86,87] demonstrating clinical improvement with etanercept, although overall the evidence is less convincing. Variation in the pharmacodynamic properties of these three drugs may partly explain the observed difference in efficacy.

## 8. Pityriasis Rubra Pilaris

Pityriasis rubra pilaris (PRP) refers to a group of uncommon idiopathic chronic inflammatory dermatoses characterised by papulosquamous eruptions. The pathogenesis is poorly understood. Immune dysregulation has been hypothesised, and PRP has been reported in association with various autoimmune conditions including hypothyroidism, diabetes mellitus, vitiligo, arthritis and dermatomyositis [88]. The histopathological features of PRP are highly variable, and show some similarities to those of psoriasis [89].

Biologics have been used successfully in the treatment of PRP, although most evidence is from case reports and small series. The demonstration of elevated levels of TNF-α in PRP lesional skin biopsies, [90] combined with the reported cases of successful treatment with infliximab [91] and adalimumab, support the use of TNF-α antagonists in the treatment of PRP. Results from a series of seven patients with treatment refractory adult-onset PRP showed complete disease remission following treatment with etanercept or infliximab. Remission was persistent following a single course of treatment in six patients with type-I PRP, although one patient with type-II PRP required a longer course of treatment and subsequently relapsed [92]. The role of interleukin (IL)-12 and IL-23 in the aetiology of PRP is not understood, however two case reports [93,94] have shown complete response of PRP to treatment with ustekinumab. This may reflect features of immunopathology in common with psoriasis.

## 9. Alopecia Areata

Alopecia areata (AA) is an immune-mediated inflammatory disease involving the hair follicles, and is a common cause of non-scarring alopecia. Clinical presentation is widely variable, ranging from patchy hair loss on the scalp to complete hair loss on the entire body (alopecia universalis). AA has been observed in association with other autoimmune disorders, in particular vitiligo and thyroid diseases [95]. Increased expression of specific human leucocyte antigen (HLA) class I (HLA-A, B and C) and class II (HLA-DR4, DR5, DR6 and DQ3) antigens and ICAM-1 on follicular epithelial cells provides evidence for the involvement of cell-mediated autoimmune mechanisms in the pathogenesis of the disease [96,97]. Inactivation of T cells can occur through stimulation of the down-regulatory cell surface molecules CTLA-4 and PD-1, with one study demonstrating an increased expression of CTLA-4 and Fas ligand in the splenic tissue of AA resistant mice [98].

The role of biologics in the treatment of AA has yet to be defined. There are several case reports of AA developing in patients receiving treatment with the TNF-α blockers adalimumab and etanercept [99]. However biologic therapies targeting T cells may provide an alternative approach to the treatment of AA. Alefacept is a lymphocyte function-associated antigen (LFA)-3-IgG1 fusion protein that targets the CD2 molecule on T-lymphocytes, preventing the co-stimulatory interaction between LFA-3 (CD58) and CD2 and thus inhibiting the activation of T-lymphocytes. Case reports have shown clinical improvement of AA in patients treated with alefacept, [100] however a randomised, double-blind trial comparing alefacept with placebo over 12 weeks, in patients with chronic and severe AA affecting the scalp, failed to observe a statistically significant difference in treatment effect [101]. Efalizumab is a humanised anti-CD11a monoclonal antibody that inhibits T cell activation and migration. Cases of severe AA successfully treated with efalizumab have been reported [102,103] although the results of a phase II double-blind placebo-controlled trial showed that treatment for three to six months with subcutaneous efalizumab had no effect on hair regrowth, or other markers of disease severity, in patients with moderate-to-severe AA [104].

## 10. Chronic Urticaria

Chronic urticaria (CU) involves cutaneous mast cell degranulation. The role of autoimmunity in the pathogenesis of CU is supported by data from a large study [105] demonstrating a high prevalence of autoantibodies and a strong association with various autoimmune conditions among patients with CU. IgG autoantibodies directed against the IgE receptor alpha subunit (anti-FcεR1alpha) and the Fc region of IgE (anti-IgE) have been identified in the sera of patients with CU, although these antibodies are not specific to CU.

Antihistamines are the standard treatment for CU, and are effective in 45%–60% of patients [106]. A promising approach to the treatment of antihistamine-refractory patients is with omalizumab, a humanised IgG1k monoclonal antibody directed against the C-epsilon 3 domain of IgE. Binding of omalizumab to IgE prevents IgE from binding to its high-affinity mast-cell receptor. The effectiveness of omalizumab in the treatment of antihistamine-refractory CU has been demonstrated in a phase I trial [107] in which 11 out of 12 patients responded to treatment (measured using mean Urticaria Activity Score, UAS) and seven patients achieved complete resolution of symptoms. In a phase II randomised, double-blind study, treatment with a single dose of subcutaneous omalizumab at doses of 300 mg and 600 mg produced greater clinical improvement compared with the placebo group (measured using UAS over seven days, UAS7) in patients with H1-antihistamine-refractory CU [108]. The response of CU to omalizumab among patients with low levels of IgE may be explained by the various IgE-independent mechanisms of action exhibited by this agent, including downregulation of various cytokines (IL-2, IL-4, IL-13 and TNF-α), induction of eosinophil apoptosis, increase in CD4+ cell activity by ATP release, and decreased expression of FcεR1 [109,110].

## 11. Atopic Dermatitis

Atopic eczema (dermatitis) is a common inflammatory skin disease, affecting 15%–20% of schoolchildren and 2%–10% of adults [111]. The pathophysiology of the condition involves immune dysregulation and epidermal-barrier disturbance. Increased production of Th2 cytokines, including interleukin (IL)-4, IL-5 and IL-13, lead to raised IgE and diminished IFN-gamma levels. Elevated Th1 and Th17 cell sub-populations have been demonstrated, although their role in the disease process in not completely understood [112]. 

Treatment with recombinant interferon and intravenous immunoglobulin have been trialled, with varying success. A randomised, double-blind, placebo-controlled trial showed that recombinant interferon-gamma (rIFN-gamma) given by daily subcutaneous injection over 12 weeks in patients with moderate to severe atopic dermatitis was well tolerated and capable of producing significant clinical improvement. Greater than 50% improvement in physicians’ overall response evaluations was achieved in 45% of rIFN-gamma treated patients and 21% of placebo-treated patients (*p* = 0.016) [113]. The efficacy and safety profile of IFN-gamma with longer-term use has been confirmed in other clinical studies, [114] and more rapid clinical improvement has been observed in patients treated with high dose (1.5 × 10^6^ IU/m^2^) subcutaneous rIFN-gamma [115]. Treatment with IFN-α 2a has produced limited clinical improvement in patients with severe atopic dermatitis, [116] whilst treatment with low or intermediate doses of IFN-α 2b has failed to demonstrate any benefit [117]. Recombinant IFN-gamma is a useful treatment option in a subset of patients with severe, unremitting atopic eczema, and there is evidence to suggest that treatment may be most effective in those patients with low blood eosinophil percentages (<9%) and IgE levels (<1,500 IU/mL) [118].

There are reports of clinical improvement in patients treated with adjunctive high-dose intravenous immunoglobulin (hdIVIg), [119] and this improvement has been observed with a concurrent reduction in CD4+ T cell numbers [120]. However, small clinical trials have failed to demonstrate any significant clinical improvement, and have shown significantly lower efficacy of IVIg compared to treatment with ciclosporin [121,122].

Efalizumab (an anti-LFA-1 monoclonal antibody) and alefacept (an LFA-3/IgG1 fusion protein) have both shown efficacy for the treatment of moderate to severe atopic dermatitis in pilot studies, and warrant further evaluation in larger clinical trials [123,124]. Promising results have also been seen with the use of rituximab, a chimeric monoclonal antibody against the B cell surface antigen CD20. Treatment with two intravenous doses of rituximab in six patients with severe atopic eczema produced significant improvement in clinical and histological parameters [125].

Therapeutic agents targeting IgE and IL-5 have been evaluated for the treatment of atopic dermatitis. Omalizumab is a monoclonal antibody that binds free and membrane-bound IgE. A pilot study in 21 patients with moderate to severe persistent allergic asthma and atopic dermatitis showed omalizumab to be an effective treatment, regardless of pre-treatment IgE levels [126]. Mepolizumab is a humanised anti-IL-5 monoclonal antibody that blocks the stimulatory activity of IL-5 on eosinophil production and maturation. In a randomised, double-blind trial, biopsies of allergen-challenged skin from 24 atopic subjects demonstrated a significant reduction in eosinophil infiltration and tenascin deposition (a marker of repair and remodelling) in those treated with intravenous mepolizumab compared to those treated with placebo [127].

Reliable evidence for the use of TNF antagonists is lacking. Sustained clinical improvement has been reported in two cases of severe, chronic atopic dermatitis treated with the TNF receptor antagonist etanercept [128]. The use of infliximab in a pilot study of nine patients with severe atopic dermatitis demonstrated significant clinical improvement with induction therapy, however the response was not maintained with further treatment [129].

## 12. Concluding Statements

There is increasing evidence to support the use of biologic agents for inflammatory and autoimmune conditions affecting the skin. Therapeutic options for many dermatological diseases are limited and unsatisfactory. Further research in this field is needed to establish the efficacy, safety and cost-effectiveness of the biological therapies currently available, and to support the development of new treatments options.
